# Direct Depth SLAM: Sparse Geometric Feature Enhanced Direct Depth SLAM System for Low-Texture Environments

**DOI:** 10.3390/s18103339

**Published:** 2018-10-06

**Authors:** Shibo Zhao, Zheng Fang

**Affiliations:** Faculty of Robot Science and Engineering, Northeastern University, Shenyang 110819, China; shibowing@gmail.com

**Keywords:** SLAM, depth vision, sparse geometric features, pose graph

## Abstract

This paper presents a real-time, robust and low-drift depth-only SLAM (simultaneous localization and mapping) method for depth cameras by utilizing both dense range flow and sparse geometry features from sequential depth images. The proposed method is mainly composed of three optimization layers, namely Direct Depth layer, ICP (Iterative closest point) Refined layer and Graph Optimization layer. The Direct Depth layer uses a range flow constraint equation to solve the fast 6-DOF (six degrees of freedom) frame-to-frame pose estimation problem. Then, the ICP Refined layer is used to reduce the local drift by applying local map based motion estimation strategy. After that, we propose a loop closure detection algorithm by extracting and matching sparse geometric features and construct a pose graph for the purpose of global pose optimization. We evaluate the performance of our method using benchmark datasets and real scene data. Experiment results show that our front-end algorithm clearly over performs the classic methods and our back-end algorithm is robust to find loop closures and reduce the global drift.

## 1. Introduction

Visual odometry is gaining importance in the field of robotics and computer vision. Recently, a number of promising results from different visual simultaneous localization and mapping (SLAM) algorithms have been presented, which provides many good solutions for six degrees of freedom (DOF) state estimation, mapping and obstacle avoidance of mobile robots. However, most of these methods mainly rely on visual features. One of its severe drawbacks is that it cannot work properly when there is not sufficient illumination or texture information such as visually degraded environments which is dark or full of fog, smoke, etc. In contrast, depth vision may overcome pose estimation failure in low-texture environments since it mainly relies on the geometric information rather than the texture information in the environment.

In recent years, with the rapid development of depth vision sensor, the perception rate and resolution of depth images have been greatly improved, which makes the depth-based visual odometry gradually attract many researchers’ attention. Several depth odometry or mapping methods have been proposed in recent years—for example, Sparse Depth Odometry (SDO) [1], SDF Tracker [2], DIFferential ODOmetry(DIFODO) [3] and Kinect Fushion [4], etc. However, since those methods are only odometry and mapping methods, which lack global map optimization, they are therefore unable to obtain global consistency trajectory in large-scale scene. Until now, the amount of research on complete depth-image based SLAM methods is relatively small. Compared with RGB images, the depth images from current depth vision sensors still have low resolution, low frame rates as well as a small field of view, and the data is still very noisy. Therefore, with current SLAM methods based on RGB [5] or 3D laser [6,7,8], it is difficult to obtain good results when directly applying on depth images.

**Figure 1:**
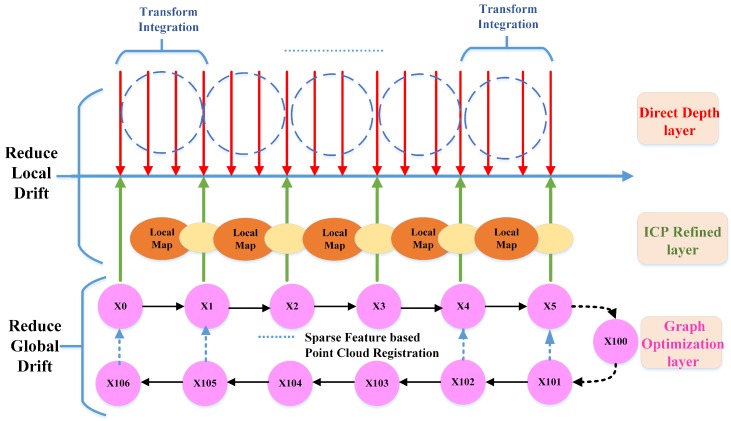
Overview of the Direct Depth SLAM (simultaneous localization and mapping) System, composed of three staggered layers, namely Direct Depth layer, ICP (iterative closet point) refined layer and Graph Optimization layer. The red arrows represent the frame to frame pose estimation of the Direct Depth layer. The transform integration module accumulates consecutive frames to construct a local map (orange ellipse in image). The green arrows represent the keyframes and their associated point clouds are denoted as yellow ellipses. The keyframes will be aligned to the constructed local map to reduce the local drift of direct depth odometry. The refined pose is stored as vertex $X_{i}$ (pink circle) in the pose graph of the Graph layer.

In this paper, we introduce a novel SLAM method, namely Direct Depth SLAM (DDS), which exploits full advantages of depth information and is able to get global consistent pose estimation in rich geometric feature environments only using 2D depth images. Since our method dosen’t rely on any RGB information, our method can estimate the motion of the camera robustly in the textureless environment. It should be noted that the definition of “textureless” here only refers to RGB images. As for depth images, we directly use the “geometric information” to describe the quality of depth images. The method has a three-layer optimization scheme, namely Direct Depth layer, ICP Refined layer and Graph Optimization layer as shown in Figure 1. This method can accurately estimate 6-DOF ego-motion in real time as well as eliminate global drift in rich geometric environments with small computation cost. Since our front-end algorithm only takes about 14 ms to estimate the ego motion between two consecutive frames ( more detail in Section 6.1.1), it can run more than 50 Hz on a normal laptop without GPU. In addition, our back-end algorithm can robustly find loop closures to reduce global drift. In order to evaluate the performance of our method, extensive experiments have been carried out. The main contributions of this paper are as follows:

- A novel depth-only SLAM scheme that utilizes both dense range flow and sparse geometry features from depth images.
- A fast direct depth odometry which runs at a high frequency to estimate frame to frame motion together with an efficient keyframe-based ICP to reduce local drift.
- An efficient geometry feature extracting and matching method to find loop closures together with a pose graph optimization to eliminate global drift.

The rest of the paper is organized as follows: Section 2 reviews various pose estimation algorithms related to the RGB-D camera. The algorithm overview will be presented in Section 3. Section 4 describes the details of the front-end algorithm. The back-end algorithm is explained in Section 5. Quantitative and qualitative evaluation of our method from front-end to back-end on publicly available datasets and the real scene are presented in Section 6. Section 7 concludes the paper.

## 2. Related Work

In this section, we first would like to introduce the current framework followed by most of the Visual SLAM algorithms since late 2010s. According to the survey papers in robotics [9,10], the relationship between Visual SLAM (VSLAM) and Visual Odometry (VO) can be represented as follows:

(1) VisualOdometry=Initialization+Tracking+Localmapping,

(2) VisualSLAM=VisualOdometry+GlobalmapOptimization.

The main difference between these two techniques is whether global map optimization is used in the mapping. In the VO, the geometric consistency of a map is considered only in a small portion of a map or only relative camera motion is computed without mapping [10]. In contrast, in the VSLAM, the global geometric consistency of a map is normally considered. Therefore, to build a geometrically consistent map, the global optimization is added in the recent VSLAM algorithms. According to the definition of VSLAM, our method is mainly composed of three modules as follows:

- Odometry method (including initialization and tracking),
- Mapping method,
- Global map optimization (including loop detection, pose graph, etc.).

**Figure 2:**
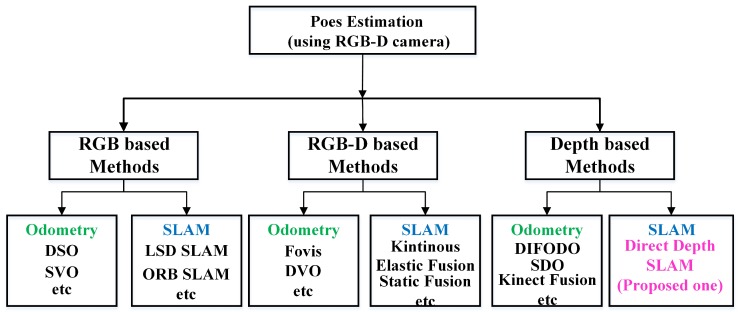
General classification of pose estimation methods based on an RGB-D camera (A RGB camera combined with a depth camera).

Currently, most pose estimation methods based on RGB-D cameras can be classified into three categories shown in Figure 2: first, RGB-based methods that usually depend on a lot of information from RGB images; second, RGB-D based methods that depend on both RGB and depth information; third, depth-based methods that only use depth data for 6-DOF motion estimation.

### 2.1. RGB-Based Methods

RGB-based methods mainly depend on RGB information, which can be divided into two sub-groups, namely RGB odometry, RGB SLAM. The first category is RGB odometry [11,12,13,14]. Those methods usually extract sparse visual features and then find features’ correspondence by matching the descriptor of visual features. After this, the Random Sample Consensus (RANSAC-based) strategies [15] are usually used to reject outliers. Then, the 3D information of visual features is calculated through triangulation. Finally, the pose estimation is calculated by minimizing the re-projection errors. There are also so-called dense visual odometry methods [16] which are different from sparse visual feature-based methods because they use the whole image to estimate the transformation. This kind of method assumes a world point observed by two cameras that is assumed to yield the same brightness in both images. The goal of these methods is to find the camera motion that best satisfies the photo-consistency constraint over all pixels. Since odometry methods only estimate the ego motion from two consecutive frames, these methods will inevitably accumulate drift. Hence, loop closure detection and back-end optimization (Pose-graph [17,18] and Bundle Adjustment [19,20]) are usually used to form a complete RGB SLAM system.

For RGB SLAM, Davison [21] presents MonoSLAM, a famous real-time algorithm which can recover the 3D trajectory of a monocular camera using an extended Kalman filter (EKF). Jakob [22] proposes a direct (feature-less) monocular SLAM algorithm that allows for building large-scale, consistent maps. Raul Mur-Artal [5] presents ORB(Oriented FAST and Rotated BRIEF)-SLAM, a feature-based monocular SLAM system that can operate in versatile environments. However, all of these methods are very sensitive to illumination and cannot robustly estimate the pose of camera in low texture scenes such as in dark environments. In contrast, since our method only depends on depth image, the texture variation of environment will not affect the accuracy of pose estimation. Qin [23] proposes a great robust and versatile monocular visual-inertial slam system. If we block the RGB input of this system for a while, its front-end algorithm can overcome low-texture environments and recover the motion of camera because of tightly-coupled IMU. However, if we block the RGB input of this system for a long time, the visual-inertial estimator can only trust the pose estimation of IMU which will produce large drift over time and its back-end optimization could also not work properly because of insufficient illumination. However, our system can still work properly because of using geometric information in the environment.

### 2.2. RGB-D Based Methods

RGB-D based methods depend on both RGB and depth information, which can be divided into two sub-groups, namely RGB-D odometry and RGB-D SLAM. Unlike the RGB odometry methods, RGB-D odometry methods [24,25,26,27] do not need triangulation but use the depth information directly from depth images. Since these odometry methods are only a kind of relative pose estimation lacking global map optimization, these methods will inevitably accumulate drift. Hence, loop closure detection and back-end optimization are also needed to form a complete RGB-D SLAM system. For RGB-D SLAM, Whelan [28] presents a real-time dense SLAM system named Kintinous which produces impressive results. However, since this approach mainly uses volumetric fusion for dense RGB-D-based tracking and mapping, the performance of this approach is deeply dependent on GPU. In addition, the loop detection of this system mainly uses RGB information to build up constraints of estimated poses. Therefore, when there is not enough texture information in the environment, the global map optimization will not work properly and the system will produce global drift over time. Endres [29] presents a 3D SLAM system that extracts visual keypoints from the color images and uses the depth images to localize them in a 3D environment. Ming [30] presents a novel keyframe-based dense planar SLAM system, which fuses depth measurements from small baseline images. However, since those methods mainly depend on visual features to achieve front-end tracking and back-end optimization, they could not work properly in a visually degraded environment. Martin [31] presents Co-Fusion, a real-time RGB-D SLAM system capable of segmenting a scene into multiple objects using motion or semantic cues. However, since this method needs to track the 6-DOF rigid pose of each active object in the current frame, its computation cost is relatively high, which leads its real-time performance to not being very good. Scona [32] proposes a method for robust dense RGB-D SLAM in dynamic environments that detect moving objects and simultaneously reconstruct the background structure. However, since this method is mainly based on GPU, its application scenarios will be limited. Compared with most RGB-D SLAM methods, since our method does not need feature matching and triangulation processes, its computation expense is relatively low, which can maintain the real-time performance even on computation limited Micro Arie Vehicles (MAVs).

### 2.3. Depth-Based Methods

For methods only using depth data from RGB-D cameras, Iterative Closest Point (ICP) [33], Generalized ICP (GICP) [34] and Normal Distribution Transform (NDT) [35] algorithms are the most classic techniques for point cloud registration. ICP and GICP are iterative algorithms that refine the initial estimate until it converges. Therefore, those methods are sensitive to initial guess and cannot cope with point clouds with large displacement. NDT models the scene with sets of small Gaussian distributions computed from the neighborhood of each point. However, the accuracy of this method depends on the size of grid. There are also some methods that apply truncated signed distance function (TSDF) [36] to describe the structure of scene and estimate the ego-motion. A famous example is KinectFusion [4], which introduces TSDF to represent structure of the scene and uses ICP to align current point cloud to the reconstructed scene model to obtain the ego-motion of camera. However, one of its limitations is that it can only be used in a small workspace. In addition, since there is no global map optimization, the KinectFusion will have drift after running for a while. Furthermore, the implementation of these methods depends on GPU.

There are also some new methods proposed in recent years working with depth cameras. Yousif [37] presents a real-time 3D registration and mapping method for texture-less scenes only using the depth information provided by a low cost RGB-D sensor. The proposed registration method is based on a novel informative sampling scheme that is able to extract the points carrying the most useful information from two consecutive frames. Taguchi [38] presents a real-time SLAM system for hand-held 3D sensors that uses both point and plane primitives for registration. Renato [39] presents a Dense Planar SLAM algorithm that identifies, merges and compresses the arbitrary planar regions. DIFferential Odometry (DIFODO) [3] derives from the concept on spatial and temporal linearization of a range function, which is similar to our front-end algorithm. Although the above methods can obtain promising results for pose estimation, those methods lack a loop detection module and can not reduce global drift in large scenes. Sparse Depth Odometry (SDO) [1] obtains the ego-motion by extracting features on depth data and matching the features to calculate the relative transform of consecutive frames. Nevertheless, since the depth image of common RGB-D camera is very noisy, sometimes it is difficult to extract reliable geometric-distinctive features when the geometric features of the scene are very sparse. Apart from this, this method is also a kind of relative pose estimation. Therefore, it lacks the ability to reduce overall drift of trajectory in large scene. SungYeon [40] proposes a keyframe-based featureless light-weight SLAM utilizing a single depth image stream. However, since the loop detection of this method is only based on a direct method, it is difficult to build loop closure constraints when there is big front-end drift. In summary, it is relatively difficult to find a complete depth only SLAM system (including odometry and global map optimization) with good performance in the literature. This paper exactly aims to achieve a complete depth only SLAM system to solve the pose estimation problem in low texture environments.

## 3. Algorithm Overview

**Figure 3:**
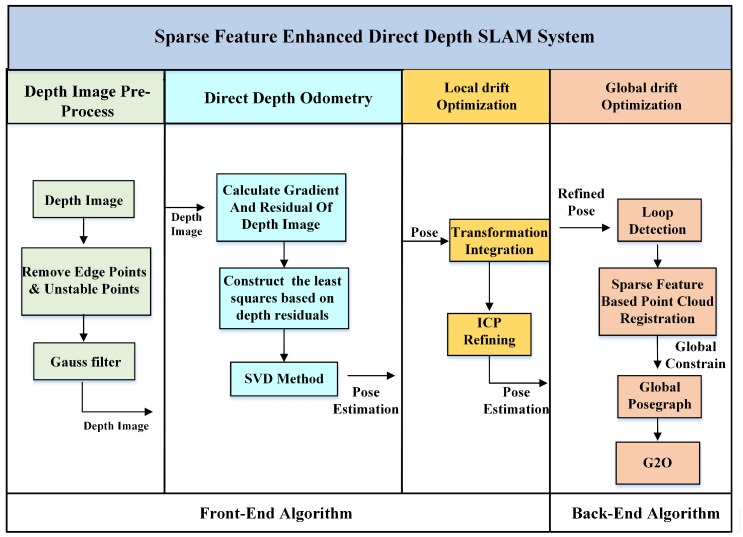
The pipeline of the proposed Depth SLAM System.

In this section, we introduce the pipeline of the direct depth SLAM System as shown in Figure 3, which can be divided into four modules: depth image pre-processing module, direct depth odometry module, local drift optimization module and global drift optimization module.

### 3.1. Depth Image Pre-Processing Module

Compared with RGB images, range data of the depth image from an RGB-D camera is usually very noisy. Since we use a range change constraint equation [41,42] (detailed in Section 4.2) to solve the motion estimation problem, we need to carefully clean up the depth data for accurate estimation. There are three kinds of noise that we must remove: isolated sparse pixels, pixels with depth value out of range, and pixels on the edge of an object. The reason is that those pixels have very unstable depth value, which will break the “local planar” assumption of range change constraint equation.

### 3.2. Direct Depth Odometry Module

After depth image pre-processing, only pixels with good depth value will be remained. Then, we use range flow constraints equation to solve the ego-motion estimation problem. The method could be divided into three steps:

- Compute the gradient of consecutive frames and calculate the depth residuals,
- Construct the least squares based on depth residuals,
- Calculate the incremental pose estimation using SVD methods.

### 3.3. Local Drift Optimization Module

Since our direct depth odometry only estimates the relative motion of camera, it will accumulate drift. In addition, the direct depth odometry assumes “small motion” assumption [41,42]. If the motion between consecutive frames is large, it will break this assumption. As a result, the accuracy of pose estimation will degenerate dramatically. For these reasons, we use a localmap based ICP method to reduce the local drift of our direct depth odometry. We first accumulate several frames of point cloud using direct depth odometry. Then, we calculate a refined pose estimation by aligning the current keyframe to the local map using ICP with a initial guess calculated from direct depth odometry. Finally, we update the odometry estimation by integrating the direct odometry with the refined pose. By doing so, our method can greatly reduce the local drift of direct depth odometry while keeping high estimation frequency.

### 3.4. Global Drift Optimization Module

Since the accumulative drift of the front-end algorithm is inevitable after a long time of running, we add a Global Drift Optimization Module to reduce global drift to get a consistent global trajectory. We first propose a very fast loop closure detection algorithm based on sparse geometric feature extracting and matching using an NDT map [35,43]. Then, we construct a pose graph to reduce the global drift by using pose graph optimization.

## 4. Front-End Algorithm

In this section, we will describe how to estimate the relative transform between two consecutive frames by using the range flow constraint equation. Then, the whole principle of direct depth odometry will be described. After that, we describe the ICP refined method for reducing local drift.

### 4.1. Depth Image Pre-Processing

Nowadays, commodity-level RGB-D cameras have a very limited measurement range and their data are also very noisy compared to laser scanners. For example, when the measurement distance is less than 3 m, usually the measurement error is less than 2.5 cm. However, when the measurement distance is at 5 m, the measurement error could be around 7 cm. In order to get accurate estimates, we first only use pixels with depth value range from 0.5 m to 4.5 m. Then, all the edge pixels and isolated sparse pixels are removed. Finally, we apply a Gaussian Filter [44] with a 7×7 kernel to smooth the depth image before calculating the depth gradients. Those steps are particularly important for our direct depth odometry algorithm to get accurate pose estimation.

### 4.2. Direct Depth Odometry Algorithm

Let a 3D point $P={\left(X,Y,Z\right)}^{T}$ (measured in the depth camera’s coordinate system) be projected on depth image $Z_{t}$ at pixel position $p={\left(x,y\right)}^{T}$. We assume the 3D point undergoes 3D motion $\Delta P={\left(\Delta X,\Delta Y,\Delta Z\right)}^{T}$ between the $t_{0}$ frame and $t_{1}$ frame, which results in a depth image motion Δp. Given that the depth value of 3D point will have moved by ΔZ, the depth value obtained at this new position *p* + Δp on image plane will have consequently varied by this amount:

(3) $$Z_{t+1}\left(p+\Delta p\right)=Z_{t}\left(p\right)+\Delta Z.$$

This equation is called a range flow constraint equation [45,46]. Taking the first-order Taylor expansion of the term $Z_{t+1}\left(p+\Delta p\right)$ in Equation (3), we can obtain

(4) $$\begin{array}{cc} Z_{t+1}\left(p+\Delta p\right) & =Z_{t+1}\left(p\right)+\nabla Z_{t+1}\left(p\right)\Delta p\\ & =Z_{t}\left(p\right)+\Delta Z, \end{array}$$

where $\nabla Z_{t+1}\left(p\right)\;$ is the gradient of depth image $\nabla Z_{t+1}\left(p\right)=\left(Z_{x},Z_{y}\right)$.

In the case of the pinhole camera model, any small 2D displacement Δp on an image plane can be related directly to the 3D displacement ΔP. We can build the relation between them through differentiating the perspective projection function in Equation (5):

(5) $$\frac{\partial p}{\partial P}=\frac{\Delta p}{\Delta P}=\left[\begin{array}{ccc} \begin{array}{c} \frac{f_{x}}{Z}\\ 0 \end{array} & \begin{array}{c} 0\\ \frac{f_{y}}{Z} \end{array} & \begin{array}{c} -X\frac{f_{x}}{Z^{2}}\\ -Y\frac{f_{y}}{Z^{2}} \end{array} \end{array}\right],$$

where $f_{x}$ and $f_{y}$ are the normalized focal lengths.

Under a small rotation assumption, if the the sensor moves with instantaneous translational velocity $v={\left(v_{1},v_{2},v_{3}\right)}^{T}$ and instantaneous rotational velocity $w={\left(w_{1},w_{2},w_{3}\right)}^{T}$ with respect to the environment, then the 3D point $P={\left(X,Y,Z\right)}^{T}$ appears to move with a velocity in Equation (6):

(6) $$\begin{array}{c} \begin{array}{cc} \frac{dP}{dt} & =\Delta P=-v-\omega \times P\\ & =\left[\begin{array}{cccccc} 0 & -Z & Y & -1 & 0 & 0\\ Z & 0 & -X & 0 & -1 & 0\\ -Y & X & 0 & 0 & 0 & -1 \end{array}\right]\xi \end{array} \end{array}$$

with respect to the sensor, where $\xi ={\left(w_{x},w_{y},w_{z},\nu_{x},\nu_{y},\nu_{z}\right)}^{T}$. Substituting Equations (5) and (6) into Equation (4), we can obtain

(7) $$\begin{array}{c} \underset{A}{\underset{︸}{\left(Z_{x},Z_{y},-1\right)\left[\begin{array}{ccc} \frac{f_{x}}{Z} & 0 & -X\frac{f_{x}}{Z^{2}}\\ 0 & \frac{f_{y}}{Z} & -Y\frac{f_{y}}{Z^{2}}\\ 0 & 0 & 1 \end{array}\right]\left[\begin{array}{cccccc} 0 & -Z & Y & -1 & 0 & 0\\ Z & 0 & -X & 0 & -1 & 0\\ -Y & X & 0 & 0 & 0 & -1 \end{array}\right]}}\xi \\ =Z_{t}\left(p\right)-Z_{t+1}\left(p\right), \end{array}\;$$

which can be rewritten as:

(8) $$A\xi =Z_{t}\left(p\right)-Z_{t+1}\left(p\right).$$

This equation generates a pixel-based constraint relating the gradient of the depth image $\nabla Z_{t+1}$ and the temporal depth difference to the unknown camera motion ξ. For the whole depth image, every single pixel satisfies the above equation. We can obtain the relative transformation matrix of pose estimation through building up the least squares between the frame $Z_{t}$ and $Z_{t+1}$. After that, we can recover the full trajectory of the depth camera.

### 4.3. Local Drift Optimization

One of the biggest advantages of our direct depth odometry is that it can calculate the pose estimation directly from the range flow constraint equation. It does not need to iterate to converge; therefore, the computation cost is very low. However, this method assumes “small motion”, which means the motion between consecutive frames should be small or the sampling frequency of the camera should be fast enough. Therefore, if the motion between consecutive frames is large, the accuracy of the pose estimation will decrease and the accumulative error will increase.

**Figure 4:**
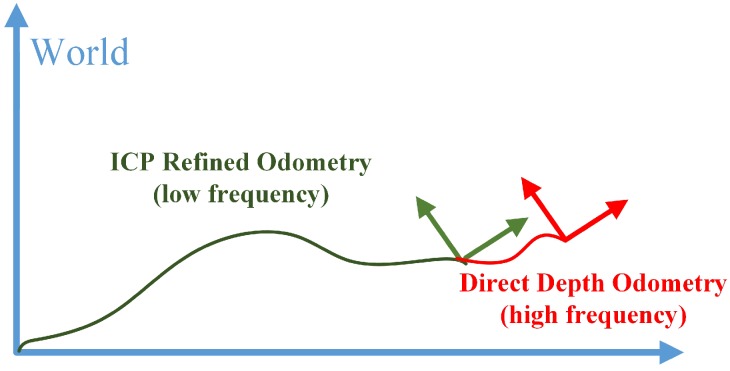
Illustration of transform integration. The green segment represents transforms published by the ICP refined odometry at a low frequency, regarding sensor poses in the world coordinate system. The red segment represents transforms published by the direct depth odometry at a high frequency. The two transforms are integrated to generate high frequency sensor pose outputs at the depth image frame rate.

To solve this problem, we use the classical ICP method to refine the pose estimation calculated from the direct depth odometry method. As shown in Figure 1, the red arrows represent the frame of pose estimation from direct depth odometry and its associated point cloud in camera coordinate. We accumulate the point cloud of every a few frames using the pose estimation from direct depth odometry to form a local map in world coordinate, depicted as an orange ellipse in Figure 1. The ICP refined layer is to align keyframes (green arrow) associated with point cloud (yellow ellipse) to the local map (orange ellipse). We accumulate the transformation estimated from direct depth odometry for the last four frames as the initial guess of the ICP registration process. Since ICP are only computed for every a few frames, we combine it with the high frequency frame to frame estimation from direct depth odometry to form the final pose estimation. As illustrated in Figure 4, the result is a high frequency integrated pose outputting at the depth image frame rate. By doing so, we keep the high frequency pose estimation of direct depth odometry as well as reduce the local drift using keyframe-based ICP.

## 5. Back-End Algorithm

The method described in Section 4.3 can only reduce local drift, and it is unable to eliminate global drift after a long time of running. In order to eliminate the global drift, we try to find loop closures and use a pose graph optimization method to remove the global drift. To achieve this aim, we need to create two kinds of constraints for the pose graph:

- **Odometry edges**: the constraint between two neighbor vertexes obtained from the ICP layer, the so-called odometry edge. In Figure 1, the pink circles in the Graph Layer represent the vertex and the black arrows represent the odometry edge built by two neighbor vertexes.
- **Loop edges**: when a loop closure is detected, a loop closure edge (dashed blue arrows) will be established, as shown in Figure 1.

In general, the establishment of odometry edge from depth images is relatively easy while loop-closure edge is much harder. There are several reasons that make the loop closure detection difficult. First, our loop-closure detection is only based on depth data. Up to now, many 3D laser SLAM systems use ICP, GICP or the NDT method to create loop edge. However, those methods are not robust on depth images since the depth cameras have a very small field of view and measurement range is very limited compared to 3D laser scanners. Therefore, when a potential loop closure occurs, usually the overlapping region of the current frame and previous frame is small, which will make those methods fail. In addition, GICP, ICP and NDT methods are sensitive to initial guess. If the drift of front-end algorithm is big, usually those methods could not find a correct transform. Therefore, it is hard to create correct a loop edge by applying those methods with depth images.

**Figure 5:**
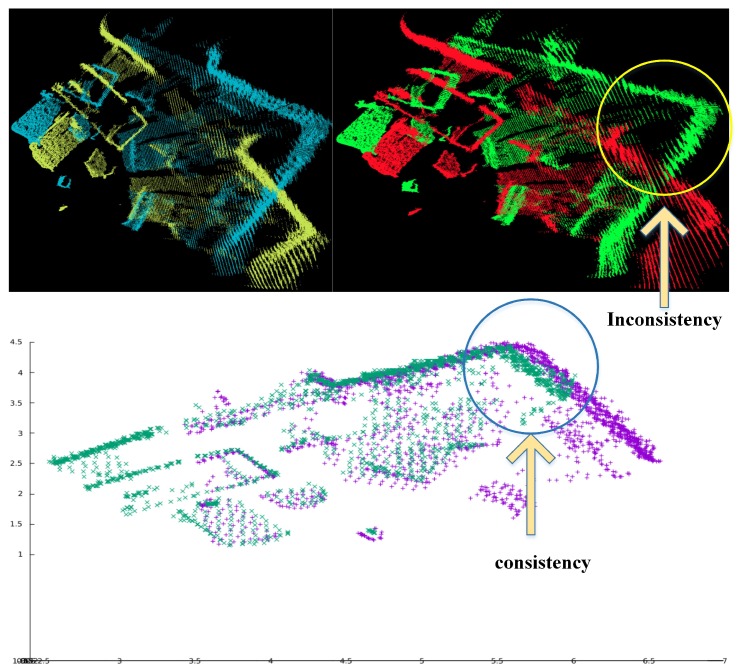
The Paired cloud before the ICP process (**top left**), the Paired cloud after ICP process (**top right**), and the Paired cloud after using sparse geometric feature matching method (**bottom**).

For example, there are two frames of point clouds with large displacement in Figure 5 (top left). If we use the ICP method, usually it is difficult to find correct estimation as shown in Figure 5 (top right). To solve the problem, we use a sparse geometric feature matching method based on the NDT map [47] to achieve real-time and robust loop closure detection [48]. The main idea is to detect local regions of salient surface curvature in the NDT map and characterize those areas with high distinction. Once all keypoints and their corresponding descriptors have been created separately for each map, they will be matched to calculate the transform. Figure 5 (bottom) shows the result of paired cloud after using sparse geometric feature matching method. We can clearly find that the pointcloud with large displacement can match correctly.

### 5.1. Feature Extraction

To extract features from the NDT-map, the following five steps are repeated for every NDT-cell $N_{i}$ within the NDT-map. Here, $N_{i}$ is the currently processed NDT-cell, called a base cell.

**Figure 6:**
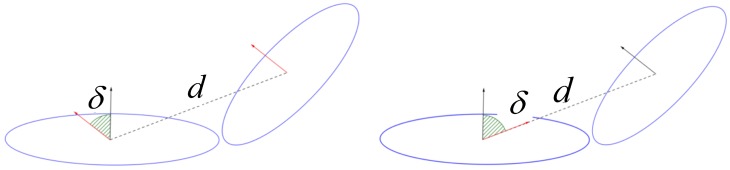
Computation of an *d* and δ between NDT(Normal Distribution Transform)-cell and base cell for matrix A (**right**) and matrix S (**left**).

- **Step 1:** Create a matrix *A* with *m* rows and *n* columns, and initialize every element to zero.
- **Step 2:** Find all neighbors $N_{k}$ of base cell $N_{i}$ within radius δ using a KD-Tree.
- **Step 3:** For each neighbor cell $N_{k}$, calculate *d* and δ, where *d* is the Euclidean distance between base cell $N_{i}$ and neighbor cells $N_{k}$, $d={∥\mu_{k}-\mu_{i}∥}^{2}$, $\mu_{i}$ and $\mu_{k}$ are the mean vectors of $N_{i}$ and $N_{k}$, δ is the smallest angle among the normal vectors of $N_{i}$ and $N_{k}$, as shown in Figure 6.
- **Step 4:** In order to use the matrix *A* to describe the geometric relationship of $N_{i}$ and $N_{k}$, we divide the largest distance *d* and angle δ into four equal divisions for different distance or angle ranges, which associates with row number (m) and column number (n) of matrix *A*, respectively. Each element $a_{ij}$ of matrix *A* represents the number of neighbor cells $N_{k}$ that both satisfy the distance range and angle range. Then, we normalize the matrix *A*.
- **Step 5:** For each NDT cell, calculate its entropy as defined in Equation (9) [47] to decide if the NDT cell can be classified into a feature category. We can compute the entropy *H* with every element in matrix A. If the entropy *H* excesses a threshold, $N_{i}$ base cell will be considered as a geometric feature:
  (9) $$\begin{array}{c} H=-\sum_{i=1}^{m}\sum_{j=1}^{n}s_{ij},\\ S_{ij}=\left\{\begin{array}{c} 0,\\ a_{ij}\log_{2}a_{ij}, \end{array}\right.\begin{array}{c} :a_{ij}=0,\\ :a_{ij}>0, \end{array}\\ H_{\max}=\log_{2}\left(mn\right). \end{array}$$

### 5.2. Feature Descriptor

After extracting the geometric features form the NDT-map, we need to create a descriptor to describe the geometric feature for the matching process. We build up the descriptor as $D=\left\{K,U,A,S\right\}$, where *K* represents the number of the neighbor cells within the radius δ of base cell $N_{i}$. *U* is the mean vector of base cell $N_{i}$. Matrix *A* is the the histogram of *d* and δ described in last section. Similarly, Matrix *S* is the histogram of *d* and the angle between normal vector of base cell and direction vector of base cell and its neighbor cell shown in Figure 6 (right). The descriptor *D* is established to represent geometric characteristics of the current geometric feature and its neighbor cells.

### 5.3. Pipeline of Loop Closure Detection

The whole pipeline of loop closure detection using the sparse geometric features is as follows:

- **Step 1:** Build an NDT-map of current frame and historical keyframes.
- **Step 2:** If the distance between current frame and any historic frame is under a threshold, we consider there is a potential loop closure.
- **Step 3:** Extract geometric features from two NDT-maps and build up their descriptor.
- **Step 4:** Using a brutal force matching method to find feature correspondences in the two frames.
- **Step 5:** Remove outliers by using the RANSAC method [15].
- **Step 6:** Compute the pose transformation matrix through the SVD method [49].

Through the above steps, our method can match two point clouds far away from each other as long as they have enough geometric features, such as the one shown in Figure 5 (bottom). You can see that this method can align the two frames very well while ICP and NDT methods may fail. However, since this method only uses a limited number of geometric features to calculate the transformation between the potential loop closure frames, the accuracy of estimation is limited. In order to improve the precision of this method, we combine the advantages of a sparse geometric feature matching method with the ICP method. The reason is that the sparse geometric feature matching method can provide a good initial guess for the ICP method and make the ICP method avoid falling into the local optimal. Meanwhile, the ICP method further improves the accuracy of the transformation estimated by a sparse geometric feature matching method. By combining these two methods, the estimated loop closure constraints are more accurate and robust, which will be added into the pose graph. For the pose graph optimization, we use g2o [50] to solve the problem.

## 6. Experiments and Analysis

To validate the performance of our proposed depth SLAM algorithm, we compared a front-end algorithm to other classic methods and tested the whole system in practical environments. The SLAM system is implemented using C++ language in ROS framework. We tested our algorithm on an Intel i7-3630 QM notebook with eight CPUs running at 2.8 GHz. Our experiment video is available at: https://youtu.be/u7aPzF0RrUc.

### 6.1. Comparison of the Front-End Algorithm

In order to show the excellent performance of our front-end algorithm, we compared our method with other classic methods (ICP, GICP, NDT) on a publicly available benchmark TUM RGB-D dataset [51].

#### 6.1.1. Real-Time Performance of the Front-End Algorithm

We found that all the tested algorithms basically satisfy real-time requirements, as indicated in Table 1. Obviously, our fronted-end algorithm is the fastest one, consuming around 14 ms for consecutive registration. The NDT algorithm is the slowest, costing nearly 32 ms. In addition, the GICP and ICP take 25 ms and 23 ms for sequential frame registration. The reason why our method is much faster than other methods is that our method can calculate the motion estimation in one single step while others need to iterate several times or extracting features.

#### 6.1.2. Accuracy Performance of the Front-End Algorithm

**Figure 7:**
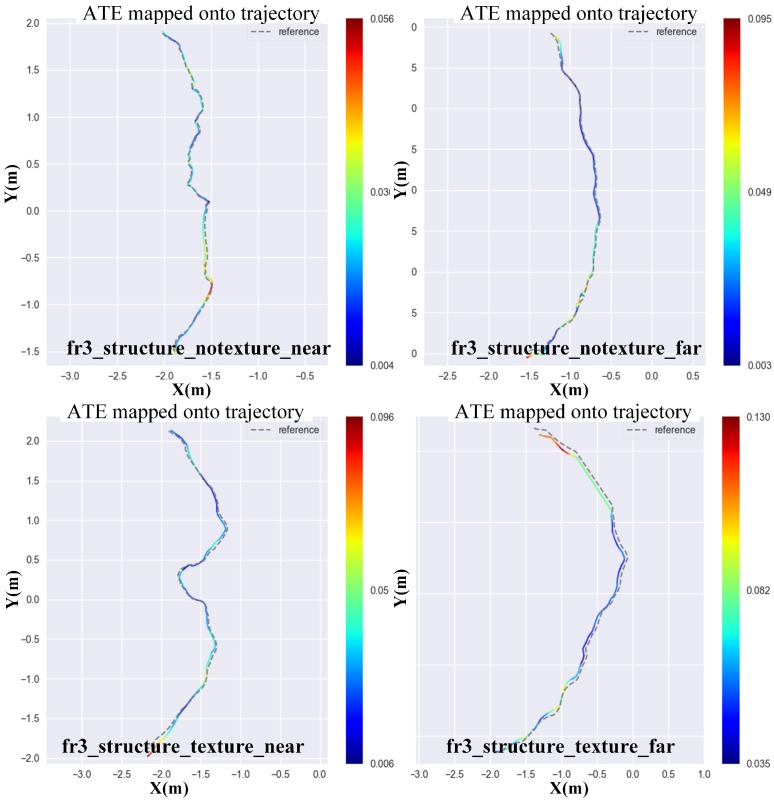
Ground truth vs. our trajectory for all kinds of datasets. The darkness of color reflects the value of ATE (Absolute Trajectory Error). The darker the color is, the smaller the value of ATE.

In this section, we use four TUM RGB-D datasets to test the estimation accuracy of each method. The dataset contains color and depth images along with ground truth trajectory that is obtained by a motion capture system. The datasets also come with evaluation routines to measure two error metrics, namely Absolute Trajectory Error (ATE) and Relative Pose Error (RPE). Although ATE only considers translational errors, any rotational error during the camera motion automatically presents as a translation error in later frames. Meanwhile, the ATE provides results that are more intuitive than those of RPE. Therefore, we use ATE to evaluate the accuracy of each method. Figure 7 shows the 2D projection of the ground truth and estimated trajectory by direct depth odometry on four datasets, namely fr3_structure_notexture_near, fr3_structure_notexture_far, fr3_structure_texture_near and fr3_structure_texture_far. All of these datasets have low-texture information, which may cause failure in pose estimation for the RGB-based method. From the results, we can see that the trajectory of the direct depth method is very accurate when the environment has enough geometric features. We also tested our front-end method on other TUM RGB-D datasets and compared it to other classic depth registration methods. The experiment results are shown in Table 2. From the results, we can see that, most of the time, our front-end method could get the best performance.

#### 6.1.3. Robustness Performance of the Front-End Algorithm

**Figure 8:**
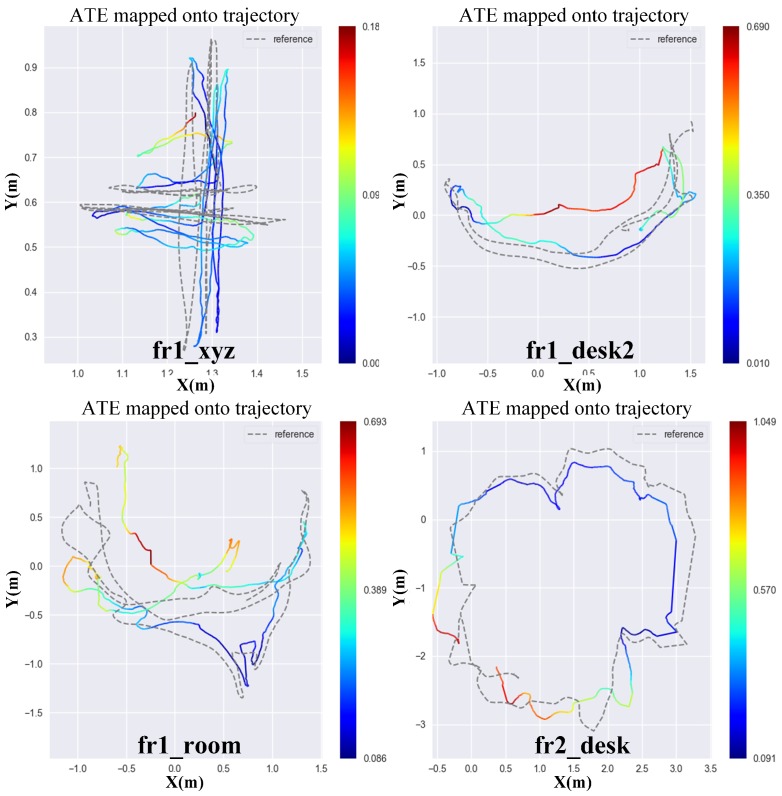
Ground truth vs. our trajectory for all kinds of datasets. The darkness of color reflects the value of ATE. The darker the color is, the smaller the value of ATE.

To test the robustness, we select clutter office scenes that are fast motion scenarios. We select fr1_xyz, fr1_desk, fr1_room and fr1_desk2. Those datasets are recorded in a typical cluster office scene with desks, computer monitors, plants, chairs, etc. In addition, the RGB-D camera in those datasets moves with fast angular velocity, and the average angular velocity of four datasets reaches 17.12 deg/s. One of the datasets, fr1_xyz, refers to a sweep motion with hand, which makes point cloud hard to match. Therefore, this is a great challenge for those geometric based methods. Figure 8 shows the estimated trajectories projected onto an *x*–*y* plane compared with ground truth. From the results, we find that the estimated trajectories have big drift in fr1_desk2 and fr1_room datasets. The potential reason is that fr1_desk2 and fr1_room datasets are relatively fast in angular velocity (23.327 deg/s and 29.88 deg/s). It breaks the small motion assumption of this method. The sampling rate is not fast enough to ensure a small relative motion between two consecutive frames. Generally, for a direct method, a fast sampling rate is good for pose estimation. Therefore, comparing with the ATE value of classic approaches on fr1_desk2 and fr1_room datasets as shown in Table 2, the accuracy of our front-end method is not very good. However, as for fr1_xyz and fr2_desk datasets, the rotation speed is 8.92 deg/s and 6.338 deg/s, respectively, which is much slower than the rest of datasets. In these two datasets, our front-end method provides better results than those of classic approaches shown in Table 2.

### 6.2. Performance of the Back-End Algorithm

#### 6.2.1. Loop Closure Detection Methods Comparison

**Figure 9:**
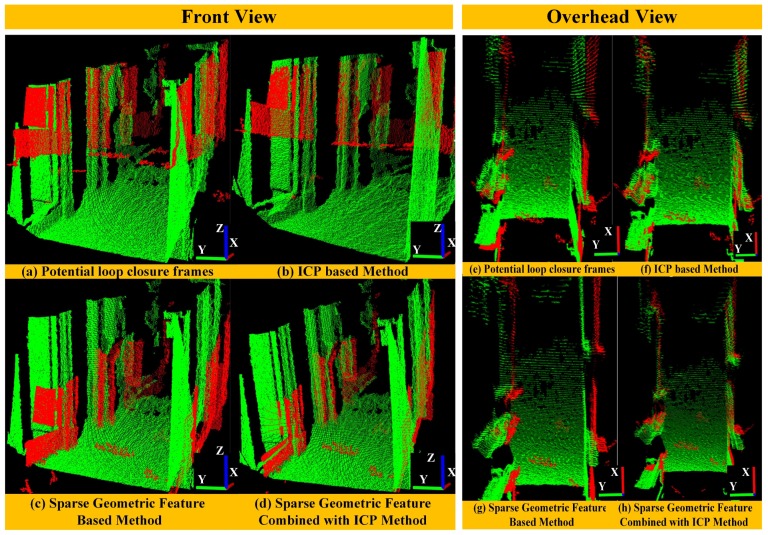
The performance of matched potential loop closure frames after applying different kinds of methods shown in the front and overhead view (The green point cloud has floor constraints, while the red point cloud does not).

**Figure 10:**
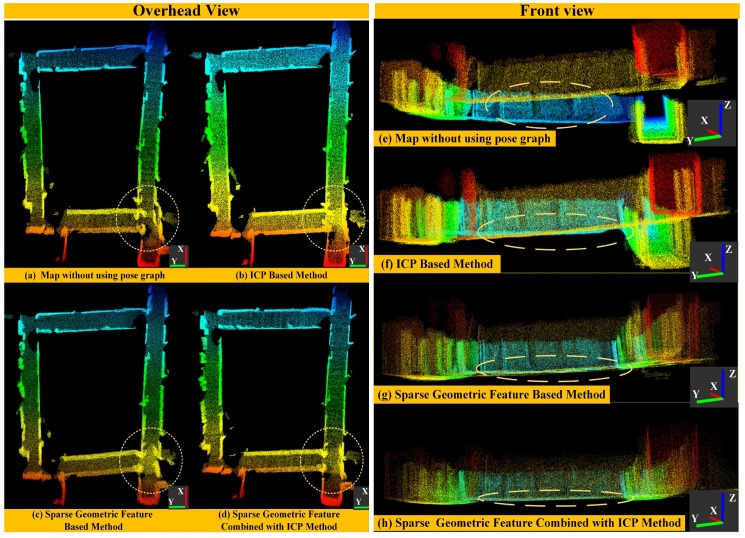
The accuracy comparison of mapping results after applying different kinds of methods for loop detection in the overhead and front view (the yellow dashed circle denotes the main difference in the global map).

(1) For Potential Loop Closure Frames: We try to find a loop closure when the distance between current keyframe pose and a historical keyframe pose is within a certain threshold. After that, we try to match the potential loop closure frames and compare the performance of different loop detection methods, namely, the ICP method, a sparse geometric feature based method and the sparse geometric feature combined with the ICP method. Figure 9 shows the results of matched potential loop closure frames after applying these methods. Since in this case there is a large accumulated error, we can clearly see large displacement between the two potential loop closure frames as shown in Figure 9a,e. The traditional ICP method falls into the local optimum easily as shown in Figure 9b,f. In contrast, the sparse geometric feature matching method can avoid the local optimum well and find the global optimal matching. However, since this method only uses a limited number of geometric features to calculate the transformation between the potential loop closure frames, the precision of estimation is not very high. As you can see from Figure 9c, the potential loop closure frames match correctly in the direction of the *z*-axis while they misalign a little bit in the *x*–*y*-axis (Figure 9g). In contrast, as shown in Figure 9d,h, a sparse geometric feature combined with an ICP method can match the potential loop closure frames correctly. This method not only avoids falling into local optimum, but also improves the matching accuracy.

(2) For Global Map: In order to validate the performance of our loop detection algorithm more intuitively, we build a global map corresponding to the scene of a long corridor environment in real time. The results are shown in Figure 10. We mainly compare the accuracy of the global map after applying the ICP method, sparse geometric feature based method and sparse geometric feature combined with ICP method, respectively (the yellow dashed circle denotes the main difference in the global map). Figure 10 presents the overhead and front view of global map in a large indoor scene. We can clearly see that the global map from the front-end algorithm is inconsistent in all axis directions (Figure 10a), especially for the direction of the *z*-axis (Figure 10e). Since the front-end has some accumulated errors, the overlap between the potential loop closure frames is relative small. Therefore, for the ICP method, it is easy to fall into local optimal and it is difficult to obtain the global consistency map. As shown in Figure 10b,f, this ICP method cannot eliminate the map distortion especially in the direction of the *z*-axis. In contrast, a sparse geometric feature method can effectively find the global optimal matching, calculate the transformation between potential loop closure frames and basically eliminate the point cloud distortion (Figure 10c), especially in the *z*-direction (Figure 10g). In spite of this, the point cloud corresponding to the floor (marked by the yellow dashed circle (Figure 10g) is thick, indicating that the precision of transformation is limited. In order to improve the precision of this method, we combine the sparse geometric features with the ICP method, which cannot only eliminate the distortion of global map, but also make the point cloud correspond to the floor more accurately, as shown in Figure 10d,h. This indicates that the precision of estimated loop closure constraints is good.

#### 6.2.2. Mapping Accuracy Comparison in a Low-Texture Environment

**Figure 11:**
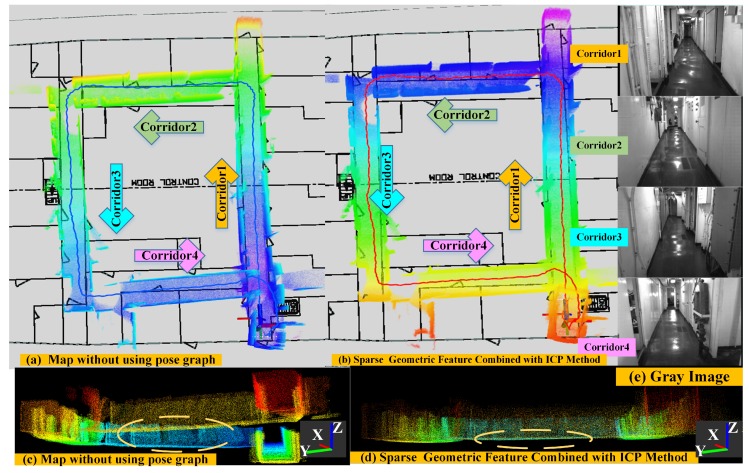
The accuracy comparison of mapping results between the front-end algorithm and back-end algorithm in overhead and front views (the yellow dashed circle denotes the main difference in global maps).

(1) Mapping Performance of Direct Depth SLAM in a long corridor environment: In order to validate the performance of our back-end algorithm, we choose a long corridor environment as shown in Figure 11e to carry out the experiments. In this long corridor, the floors and walls are very smooth. Therefore, this environment is very challenging for depth-only methods. The main challenges are:

- The walls and floor of long and narrow corridors are relatively smooth, and have less geometrical-distinctive features.
- The depth camera has a very limited measurement range and field of view; therefore, there are a number of pixels in the depth image that will have NAN depth value. Given this, it is difficult to obtain constraints along the forward direction for depth cameras in such long and narrow corridors.
- As this corridor environment covers 20 m × 30 m, the accumulated drift of the front-end method should be small enough. Otherwise, the loop closure constraint at the back-end algorithm will be hard to establish. Therefore, this requires the pose estimation of the front-end algorithm to be highly precise.
- The corridor environment is also very difficult for extracting geometric features since it only contains smooth walls. Therefore, it is difficult for the back-end algorithm to determine previously visited places.

The experimental results are presented in Figure 11. The background is the floor plan of the corridor environment. The camera moved along the arrows in an anti-clockwise direction. The blue line (Figure 11a) presents the trajectory estimated with our front-end algorithm. We can clearly see that the global map from the front-end algorithm is inconsistent a little bit in all directions (Figure 11a), especially in the *z*-direction (Figure 11c). In contrast, the sparse geometric feature combined with the ICP method can effectively find the global optimal matching shown as in Figure 11b,d. The red line in the (Figure 11b) shows the trajectory of pose estimation after global pose graph optimization, which coincides with the floor plan well. This shows that our depth image-based SLAM system has the ability to reduce the global drift in a low-texture scene by robust loop-closure detection and global pose graph optimization.

**Figure 12:**
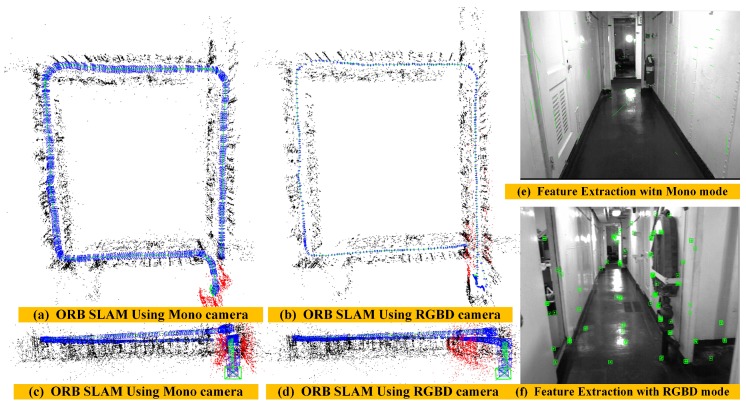
The performance of mapping results after applying the ORB-SLAM algorithm using the mono and RGB-D camera, respectively.

**Figure 13:**
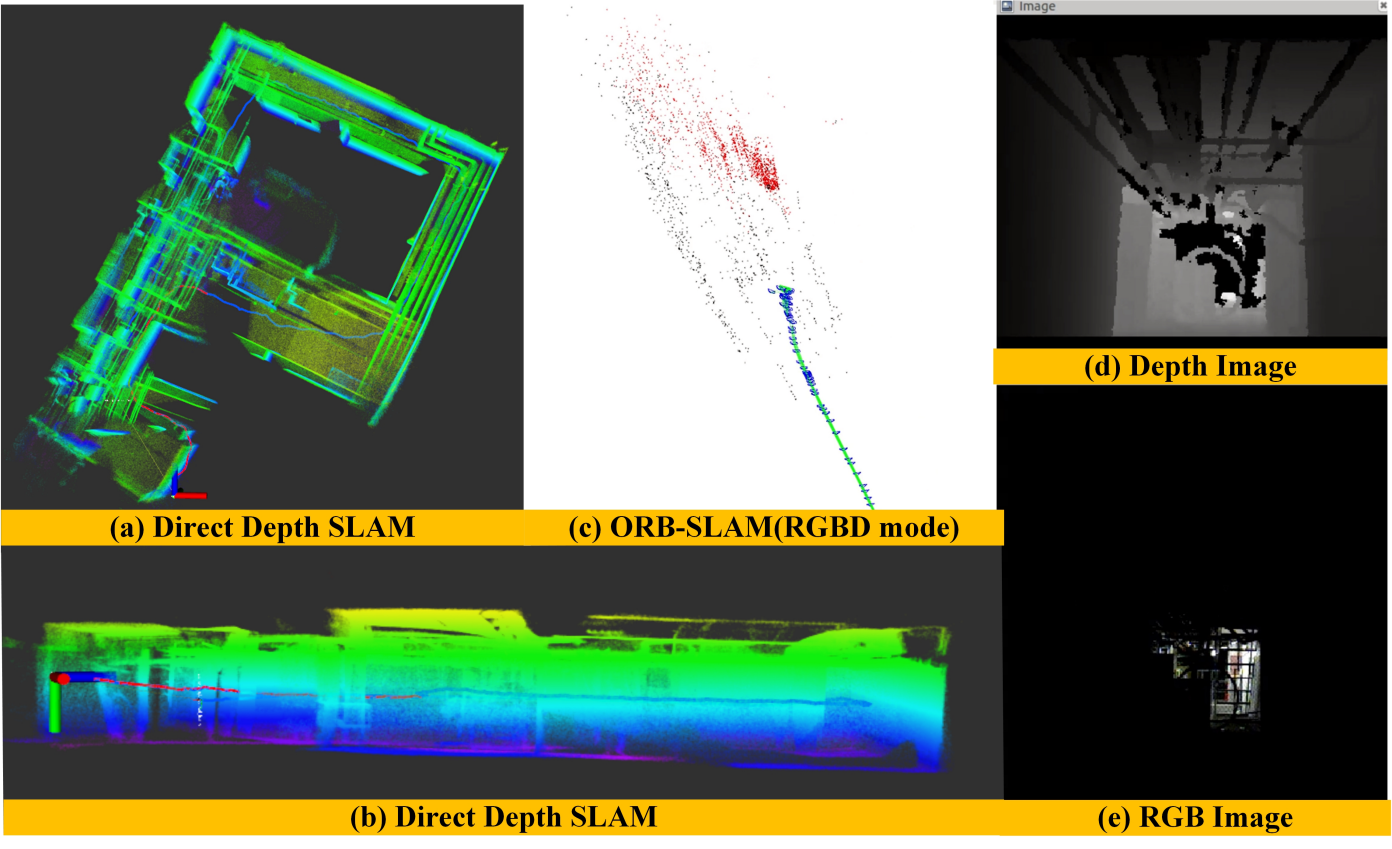
The performance of mapping results in a dark environment after applying direct depth SLAM (**a**) and ORB-SLAM (**c**) methods, respectively.

(2) Mapping Performance of ORB-SLAM in a long corridor environment: To further illustrate the performance of our algorithm, we compare our experimental results with the ORB-SLAM algorithm using the same corridor dataset. Since we cannot obtain the ground truth of this dataset, we can only evaluate the algorithm by comparing the loop closure error and the quality of the global map. Here, we use the monocular and the RGBD mode of ORB SLAM algorithm to construct the global map as shown in Figure 12. When only providing RGB information to the ORB-SLAM system, since the surfaces of the wall and floor have low texture information, the ORB-SLAM system under the monocular mode is often difficult for extracting the salient features, resulting in the system initialization and tracking failure, as shown in Figure 12e. Figure 12a,c show the global map results when ORB-SLAM initializes successfully in monocular mode. We can see that there is failure in loop detection, drift in its estimated trajectory, and distortion in its reconstructed global map. When providing both RGB image and depth image to the ORB-SLAM system at the same time, however, this system can complete the initialization work normally, and the extracted texture features of a single frame are very sparse as shown in Figure 12f. It makes the pose estimation not very accurate and also leads to the loop detection failure and distorts the global map as shown in Figure 12b,d. We can see that there is an accumulated drift in the vertical direction of estimated trajectory (Figure 12d). This experiment result fully validates the advantages of our Direct Depth Slam System for low-texture environments.

(3) Mapping Performance Comparison of different methods in a dark environment: In order to validate the robustness of our method, we choose a cluttered and dark environment to carry out the experiments. In such scenes, we can hardly obtain any texture information as shown in Figure 13e. In contrast, the depth images can provide abundant geometric information as shown in Figure 13d. Since the whole environment is very messy, it is also very challenging for depth-only methods. Figure 13 shows the performance of mapping results in a dark environment after applying our method and ORB-SLAM method, respectively. We can clearly see that our method can estimate the motion of cameras robustly in a dark environment (Figure 13a) and there is almost no drift in the vertical direction (Figure 13b) while the ORB-SLAM with RGB-D mode can not work properly(Figure 13c). The reason is that the ORB-SLAM mainly depends on texture information and does not make the depth images to form an independent pose estimation module. Therefore, when the environments lack texture information, the front-end module of ORB-SLAM cannot extract salient features and pose estimation will fail even though the depth images are available.

## 7. Conclusions

In this paper, we introduced a novel depth SLAM method that utilizes both dense range flow and sparse geometry features from sequential depth images. The proposed method is composed of three optimization layers, namely the Direct Depth layer, ICP refined layer and Graph Optimization layer. By using the proposed three-layer optimization scheme, our SLAM method can achieve real-time and low drift pose estimation. Quantitative and qualitative experimental results show that our method can achieve real-time, low drift and robustness ego-motion estimation. In the future, we will carry out more experiments to validate the performance of our method and try to extend this method to a 3D laser scanner. Especially for the upcoming solid-state laser scanner, we believe that this method will have broad application prospects. We also want to fuse our front-end algorithm with an IMU to improve its robustness.

## Figures and Tables

**Table 1:** Average registration time for each algorithm (ms) (The blue number highlights the best results for each specific dataset among all of the compared algorithms).

Datasets	GICP	ICP	NDT	Ours
fr1_360	25	22	32	15
fr1_desk2	31	27	15	12
fr1_room	21	21	27	15
fr1_xyz	29	20	38	10
fr2_desk	33	33	44	18
fr2_large_with_loop	24	23	25	16
fr3_cabinet	27	18	26	17
fr3_large_cabinet	17	19	19	15
fr3_long_office_household	20	18	20	10
fr3_structure_notexture_near	17	21	77	18
fr3_structure_notexture_far	20	18	19	17
fr3_structure_texture_near	20	17	27	17
fr3_structure_texture_far	30	29	48	8
fr3_sitting_xyz	30	23	27	13
Average time	25	23	32	14

**Table 2:** Evaluation of GICP, ICP, NDT and ours on tum datasets (The blue number highlights the best results for each specific dataset among all of the compared algorithms).

	ATE (in m) Transl. RMSE				ATE (in m) Transl. MAX			
Datasets	GICP	ICP	NDT	OURS	GICP	ICP	NDT	OURS
fr1_360	0.184	0.186	0.189	0.174	0.336	0.332	0.316	0.349
fr1_desk2	0.431	0.27	0.777	0.35	1.252	0.615	1.144	0.69
fr1_room	0.299	0.178	0.907	0.376	0.698	0.365	1.524	0.693
fr1_xyz	0.085	0.106	0.188	0.068	0.152	0.189	0.368	0.188
fr2_desk	1.155	0.632	1.557	0.533	2.136	1.356	2.284	1.049
fr2_large_with_loop	1.33	1.15	1.22	0.71	2.85	2.02	2.63	1.8
fr3_cabinet	0.555	0.458	4.718	0.356	1.208	1.004	8.529	0.856
fr3_large_cabinet	0.788	1.577	2.18	0.46	1.993	2.787	4.487	1.207
fr3_long_office_household	0.786	0.857	1.306	0.95	1.852	2.301	2.727	2.093
fr3_structure_notexture_near	0.133	0.135	0.633	0.025	0.314	0.299	1.413	0.056
fr3_structure_notexture_far	0.134	0.064	0.625	0.04	0.253	0.223	1.504	0.095
fr3_structure_texture_near	0.112	0.162	0.687	0.36	0.216	0.348	1.453	0.096
fr3_structure_texture_far	0.203	0.141	2.259	0.07	0.414	0.237	4.213	0.13
fr3_sitting_xyz	0.287	0.288	0.283	0.147	0.5414	0.599	0.568	0.2893
